# Characteristics and Clinical Outcomes in Overhead Sports Athletes after Rotator Cuff Repair

**DOI:** 10.1155/2017/5476293

**Published:** 2017-06-15

**Authors:** Tomoyuki Muto, Hiroaki Inui, Hiroki Ninomiya, Hiroshi Tanaka, Katsuya Nobuhara

**Affiliations:** Department of Orthopaedic Surgery, Nobuhara Hospital and Institute of Biomechanics, 720 Haze, Issai-cho, Tatsuno-shi, Hyogo-ken 679-4017, Japan

## Abstract

Rotator cuff tears in young overhead sports athletes are rare. The pathomechanism causing rotator cuff tears in young overhead athletes is different from that in aged patients. The purpose of this study was to investigate rotator cuff tear characteristics in young overhead sports athletes to reveal the pathomechanism causing these injuries. This study included 25 overhead sports athletes less than 30 years old with atraumatic rotator cuff tears necessitating repair. Rotator cuff tear characteristics were evaluated intraoperatively, including rotator cuff tear shape and injured rotator cuff tendon. Clinical outcome measures were assessed before surgery and at the final follow-up. In this study, 22 patients reported minimal to no shoulder pain and returned to sports without significant complaints at last follow-up. The isolated infraspinatus tendon was most often injured; the incidence rate of the tear at this site was 32% (8 cases). In the deceleration phase of overhead motion, the eccentric contraction force of the ISP (infraspinatus) tendon peaks and the increased load leads to injury at the ISP tendon. The pathomechanism of rotator cuff injuries in young overhead athletes might be not only internal or subacromial impingement, but also these mechanisms.

## 1. Introduction

Overhead shoulder injuries are variously named overused syndrome, rotator cuff injury, articular labrum damage, little league shoulder, and rotator cuff tear. Basic overhead motions such as throwing are routine, and injury develops due to repeated motion and environmental and individual factors. The reasons these injuries occur include functional disorders of the lower limbs, the chest, pelvic girdle, or the shoulder girdle and collapse of the kinetic chain. Rotator cuff tears are a common cause of shoulder injury and lead to taking a long-term break from the sport.

However, rotator cuff tears generally affect patients older than 40 years old [1]. Age-related changes reduce the mechanical properties of the rotator cuff and prompt these injuries to occur in older patients [2]. Hawkins et al. reported only 2 cases of patients younger than 40, out of 100 patients who underwent rotator cuff tear repair [3]. Neer claimed that 95% of rotator cuff tears were caused by impingement, and this condition generally occurred in patients 40 years and older [4].

Generally the cause of rotator cuff tears in young patients is trauma or overuse [5]. The repetitive nature of throwing in overhead athletes places physiological loads of up to 108% of body weight [6] and humeral angular velocities upwards of 7,000 degrees/s [7]. Because of the relatively avascular tendon insertion site, these loads and torques, which are stressed during the acceleration and deceleration phases of the throwing cycle, can lead to repetitive microtrauma to the tendon [8].

The main difference in rotator cuff tears between young athletes and older patients may be related to differences in the pathomechanism of the tear. However, little is known about the pathomechanism of rotator cuff tears in young overhead athletes.

The purpose of this study was to investigate rotator cuff tear characteristics and clinical outcomes after rotator cuff repair and provide the pathomechanism of rotator cuff tears in young overhead sports athletes.

## 2. Material and Methods

IRB approval was obtained for this study (Nobuhara Hospital IRB number 170).

### 2.1. Patient

In our hospital, 3696 patients underwent rotator cuff repair between 1980 and 2008. Twenty-five patients (mean age 22.8 years, ranged from 13 to 30 years) were treated with rotator cuff repair between 1980 and 2008. When conservative treatments, such as rehabilitation, injection, and medication, were ineffective and complaint persisted for three months after injury, surgical repair was recommended. The study group included 23 males and 2 females. All cases were dominant shoulders (right: 22 shoulders, left: 3 shoulders). The inclusion criteria were patient age younger than 30 years without trauma and rotator cuff tear necessitating repair. Exclusion criteria were patient age 30 years and older, greater tuberosity fractures, lesser tuberosity fractures, and insufficient follow-up. The mean follow-up period was 24.4 months. Diagnosis was based on history and clinical findings together with the results of arthrographic or magnetic resonance imaging (MRI) investigations.

### 2.2. Surgical Procedure and Postoperative Treatment

Under general anesthesia, in the beach chair position, a supine position was the setting. A 6 cm longitudinal incision was made lateral to the coracoid process. Blunt separation of the deltoid muscle fibers was performed and the bursa was opened to identify the rotator cuff tendon. Adhesion of the subacromial and subdeltoid was detached carefully by inserting finger or sponge. Acromionectomy was done, if there was osteophyte in the anterior region of the acromion. Nonabsorbable suture was placed on the tendon about 1 cm from the end and passed through the tendon. Bone groove was made at the central side of the humeral head. The sutures in the tendon were pulled outward thorough the bone groove [9] (McLaughlin method). The shoulder was immobilized for 2 to 4 weeks by sling or traction. Elbow and wrist range of motion exercises are begun almost immediately after surgery. Passive range of motion was performed during 2–4 weeks postoperatively, followed by active range of motion during 6–8 weeks postoperatively. Progressive strengthening was started at 8–12 weeks. This protocol was modified as needed based on the size of the tear, the patient's response to rehabilitation, and the nature of any concurrent procedures.

### 2.3. Outcomes Assessment and Data Collection

Rotator cuff tear characteristics were also evaluated in surgical findings and postoperative record: rotator cuff tear shape and injured rotator cuff tendon. The shape of the tear was measured during the surgical procedure and was based on the Nobuhara classification [10]. Incomplete tear was classified as concealed tears (articular-side tear and intratendinous tear). Complete tear was classified as anterior tears, longitudinal tear, and transverse tear. The injured tendons, such as supraspinatus (SSP), infraspinatus (ISP), and subscapularis (SSC), were also evaluated.

Clinical outcome measures were used before surgery and at the final follow-up. The shoulder function was assessed primarily using the University of California, Los Angeles (UCLA) shoulder score. The scoring system is a combination of physical exam findings (active forward elevation and strength) and subjective patient reported measures (pain, satisfaction, and function). Pain and function are preferentially weighted (20 out of 35 possible points). A higher score indicates better function. The scoring system of the Japanese Orthopaedic Association (JOA score) was also evaluated. The JOA score is a 100-point scoring system, with 30 points for pain, 20 for function, 30 for ROM, and 20 for radiographic findings and stability.

### 2.4. Statistical Analysis

Differences in gender, number of tear shapes, number of torn tendons, UCLA score, and JOA score preoperatively and at final follow-up were determined using the paired *t*-test and all analyses were performed using Stat View for Windows Version 5.0 (SAS Institute Inc., Cary, NC).

## 3. Results

### 3.1. Sports History

**Figure 1:**
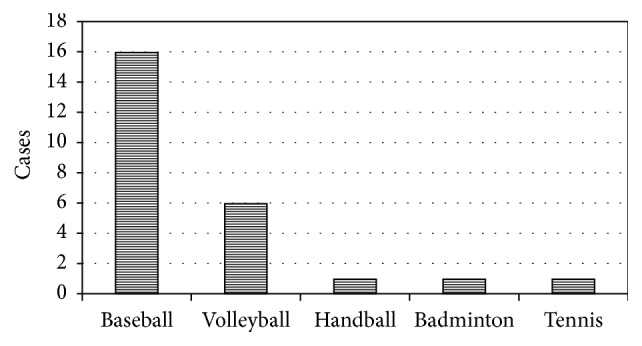
*Sports activity*. Cause of sports activity was 16 being baseball players, 6 volleyball players, 1 handball player, 1 badminton player, and 1 tennis player.

Cause of sports activity varied, including 16 being baseball players, 6 volleyball players, 1 handball player, 1 badminton player, and 1 tennis player (Figure 1). All patients were nonprofessional players.

### 3.2. Tear Characteristics

**Figure 2:**
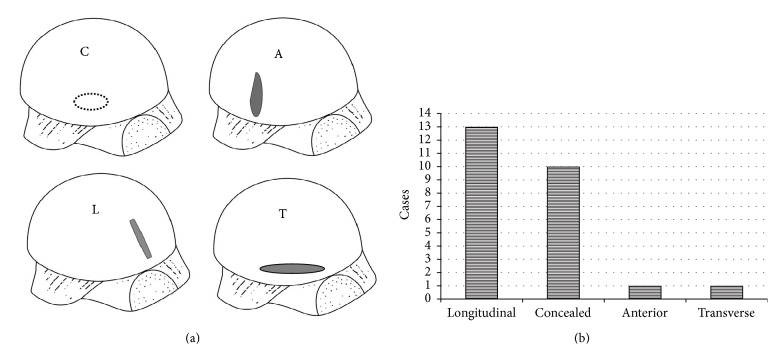
*Tear characteristic*. (a) Tear characteristic schema. C; concealed tear, A; anterior tear, L; longitudinal tear, T; transverse tear. (b) Concealed tear was found in 10 cases. Complete tear was found in 15 cases; longitudinal tear was found in 13 cases, anterior tear was found in one case, and transverse tear was found in one case.

Concealed tear was found in 10 cases (40%). Complete tear was found in 15 cases (60%); longitudinal tear (L) was found in 13 cases, anterior tear (A) was found in one case, and transverse tear (T) was found in one case. Longitudinal tear in which this tear occurred along the torn tendon fiber and the tear expanded tended to be popular in this study (Figure 2).

### 3.3. Injured Tendon

**Figure 3:**
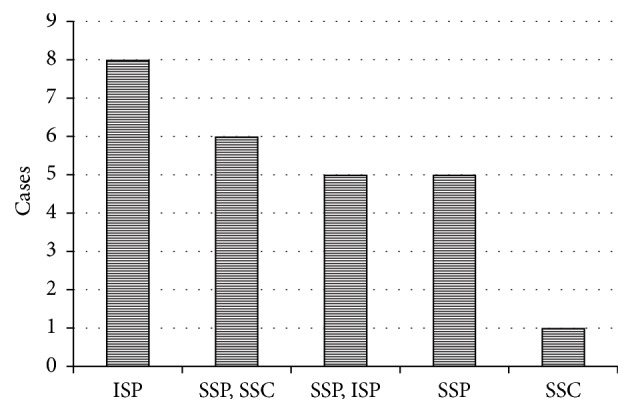
*Injured tendon*. ISP tendon was injured most frequently, followed by SSP + SSC tendon, SSP + ISP tendon, SSP tendon, and SSC tendon.

It was found that ISP tendon was injured most frequently, followed by SSP + SSC tendon, SSP + ISP tendon, SSP tendon, and SSC tendon (Figure 3) (ISP: 8 cases, SSP + SSC: 6 cases, SSP + ISP: 5 cases, SSP: 5 cases, and SSC: 2 cases).

### 3.4. Clinical Outcomes

**Figure 4:**
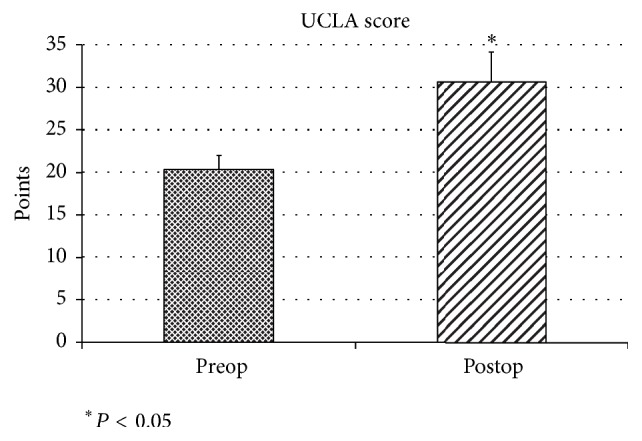
*UCLA score*. At the last follow-up, the UCLA score improved from the preoperative mean of 20.4 points to 30.6 points. There was significant difference between preoperative and last follow-up score in the JOA score (*P* < 0.01).

**Figure 5:**
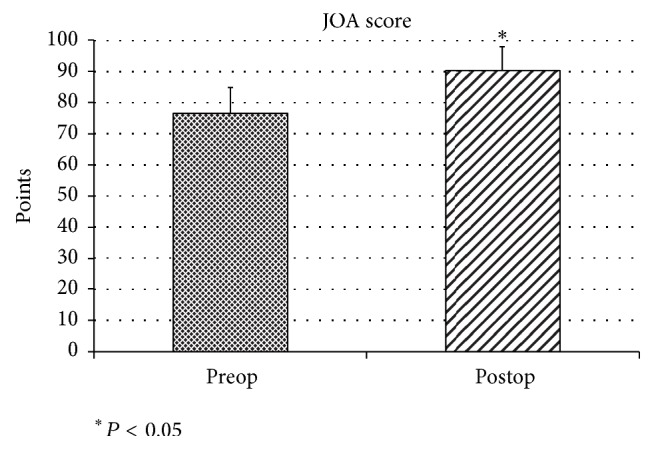
*JOA score*. At the last follow-up, the JOA score improved from the preoperative mean of 76.4 points to 90.2 points. There was significant difference between preoperative and last follow-up score in the JOA score (*P* < 0.01).

At the last follow-up, the UCLA score improved from the preoperative mean of 20.4 points to 30.6 points and the JOA score improved from the preoperative mean of 76.4 points to 90.2 points. There was significant difference between preoperative and last follow-up score in UCLA score and JOA score (*P* < 0.01). Infection or recurrent partial or total tear did not occur in this study. 22 patients had no shoulder pain at final follow-up and rate of return to their preoperative activity level after rotator cuff repair was 88% at final follow-up (Figures 4 and 5).

## 4. Discussion

The pathology of pitching shoulder disorders in baseball players is often reported to be rotator cuff injury as a result of overuse and internal impingement, and injuries of the supraspinatus muscle and/or ISP muscle and superior articular labrum anterior and posterior lesions are particularly common [11]. Internal impingement is caused by joint instability, and the physiological phenomenon that occurs when the shoulder joint is in the external rotation abduction position is as follows: the rotator cuff joint surface becomes jammed in the space between the rotator cuff insertion at the greater tubercle and the articular labrum positioned at the superior edge of the joint fossa [12]. Articular-sided tears are generally more common than bursal side tears in athletes who use overhead movements [13, 14], and a cohort study reported that 91% of partial tears in young people are articular-sided tears [15]. Articular-sided tears are probably multifactorial; factors include the relative hypovascularity of the articular cuff, due to a lower stress to failure ratio on the articular side with less distinct and randomly oriented collagen bundles and strength compared with the bursal side [16, 17]. However, Kim and McFarland showed that 277 (74%) of 376 patients had internal impingement in flexion. There were no statistically significant differences in the prevalence of internal impingement in flexion according to the primary diagnoses [18]. In this study, the most common injured cuff was the ISP tendon, not the supraspinatus, and the most common tear shape was a longitudinal tear, not an articular-side tear. Therefore, other factors might be involved in the mechanism causing rotator cuff tear in young overhead sports athletes.

First, the eccentric contraction force of the ISP tendon peaks in the deceleration phase of overhead motion. As a result, the increased load during this phase is thought to lead to injury at the insertion of the ISP muscle. The excessive anterior displacement of the humerus head during the external rotation phase might amplify the tension of the ISP muscle insertion during the internal rotation phase and increase the eccentric contraction load. Anterior instability of the shoulder joint caused by the structural shoulder injury or overhead motion from unreasonable postures increases the load on the ISP tendon. This mechanism can cause enthesitis and partial tendon tears and progress to chronic synovitis and osteochondral lesions. Conversely, the ISP muscle plays a role in inhibiting anterior deviation of the humerus head during abduction and external rotation phase. ISP tendon tear promotes anterior instability during abduction and external rotation phase, which might exacerbate the ISP tendon injury. In this study, a rotator interval (RI) lesion often coexisted with a rotator cuff tear. It seemed that repeated injury to the RI due to excessive overhead motions might lead to anteroinferior instability. This instability might cause the ISP to tear.

Secondly, the ISP muscle atrophy occurs commonly in overhead sports athletes [19]. In previous study, atrophy of the ISP muscle in athletes is caused by entrapment of the suprascapular nerve. Degeneration of the nerve in ISP muscle atrophy in volleyball players led to diagnosis with electromyogram [20]. Based on these findings, that ISP muscle atrophy is caused not only by nerve entrapment, but also by traction on the suprascapular nerve. These results suggest that atrophy of the ISP muscle may develop into ISP tendon tear. The cause of rotator cuff injuries in the young overhead sports athlete might be not only subacromial or internal impingement, but also these mechanisms.

Previous study showed rotator cuff tears most commonly involved a posterior location, near the junction of the SSP and ISP [21], and, in the longitudinal tear models, stress concentration was seen in the posterior torn tendon edge as well as the bottom of the longitudinal tear [22]. The areas with high stress concentration increased with cuff tear size. Longitudinal tears were common in this study. To prevent increasing the cuff tear size and return to the sports early with no complaint, surgery should be considered as an effective option for treating rotator cuff tears in young overhead sports athletes.

In clinical settings, the treatment for rotator cuff tears depends upon the severity of the symptoms and there are varieties of treatment options. Conservative treatment includes rehabilitation, injections, and drug therapy [23]. Even though most tears cannot heal on their own, satisfactory function can often be achieved with nonsurgical treatment for overhead sports athletes. However, conservative treatment might require long-term therapy and during that period the athlete will be forced to take a long-term break from the sport. The overall rate of return to sports after rotator cuff repair was 84.7%, including 66.9% at an equivalent level of play, 4 to 17 months after surgery [24]. In this study, 22 patients had no shoulder pain at final follow-up, and the rate of return to sports after rotator cuff repair was 88% at final follow-up. Rotator cuff tears in young overhead athletes respond well to rotator cuff repair, as shown by good patient outcomes, and high satisfaction is seen postoperatively.

## 5. Conclusions

The infraspinatus was the most commonly injured tendon in young adults (<30 y) presenting with nontraumatic rotator cuff injury. A longitudinal tear was the most common type of tear. Rotator cuff tears in young overhead sports athletes respond well to surgical repair, demonstrated by a 88% return to sport at the final follow-up (~24 months).
